# Coordination of CcpA and CodY Regulators in Staphylococcus aureus USA300 Strains

**DOI:** 10.1128/msystems.00480-22

**Published:** 2022-11-02

**Authors:** Saugat Poudel, Ying Hefner, Richard Szubin, Anand Sastry, Ye Gao, Victor Nizet, Bernhard O. Palsson

**Affiliations:** a Department of Bioengineering, University of California San Diego, San Diego, California, USA; b Department of Biological Sciences, University of California San Diego, San Diego, California, USA; c Collaborative to Halt Antibiotic-Resistant Microbes (CHARM), Department of Pediatrics, University of California San Diego, San Diego, California, USA; d Skaggs School of Pharmacy and Pharmaceutical Sciences, University of California San Diego, San Diego, California, USA; University of Massachusetts Medical School

**Keywords:** network modeling, *Staphylococcus aureus*, gene regulation, metabolism

## Abstract

The complex cross talk between metabolism and gene regulatory networks makes it difficult to untangle individual constituents and study their precise roles and interactions. To address this issue, we modularized the transcriptional regulatory network (TRN) of the Staphylococcus aureus USA300 strain by applying independent component analysis (ICA) to 385 RNA sequencing samples. We then combined the modular TRN model with a metabolic model to study the regulation of carbon and amino acid metabolism. Our analysis showed that regulation of central carbon metabolism by CcpA and amino acid biosynthesis by CodY are closely coordinated. In general, S. aureus increases the expression of CodY-regulated genes in the presence of preferred carbon sources such as glucose. This transcriptional coordination was corroborated by metabolic model simulations that also showed increased amino acid biosynthesis in the presence of glucose. Further, we found that CodY and CcpA cooperatively regulate the expression of ribosome hibernation-promoting factor, thus linking metabolic cues with translation. In line with this hypothesis, expression of CodY-regulated genes is tightly correlated with expression of genes encoding ribosomal proteins. Together, we propose a coarse-grained model where expression of S. aureus genes encoding enzymes that control carbon flux and nitrogen flux through the system is coregulated with expression of translation machinery to modularly control protein synthesis. While this work focuses on three key regulators, the full TRN model we present contains 76 total independently modulated sets of genes, each with the potential to uncover other complex regulatory structures and interactions.

**IMPORTANCE** Staphylococcus aureus is a versatile pathogen with an expanding antibiotic resistance profile. The biology underlying its clinical success emerges from an interplay of many systems such as metabolism and gene regulatory networks. This work brings together models for these two systems to establish fundamental principles governing the regulation of S. aureus central metabolism and protein synthesis. Studies of these fundamental biological principles are often confined to model organisms such as Escherichia coli. However, expanding these models to pathogens can provide a framework from which complex and clinically important phenotypes such as virulence and antibiotic resistance can be better understood. Additionally, the expanded gene regulatory network model presented here can deconvolute the biology underlying other important phenotypes in this pathogen.

## INTRODUCTION

Metabolism plays an integral role in infection and antimicrobial resistance (AMR) in the leading human bacterial pathogen Staphylococcus aureus. Metabolic requirements specific to infection, intracellular persistence, biofilm formation, and colonization are rapidly being uncovered (1–6). Furthermore, the central role of metabolism in AMR and persistence is also coming into view, adding to the complexity of known AMR mechanisms (7–9). The complex metabolic circuits and responses underlying these phenomena are nevertheless difficult to unravel. Even relatively well-understood systems such as S. aureus central carbon metabolism can be difficult to fully map, as they are layered with multiple levels of gene regulation, posttranslational and biochemical controls, and unexpected molecular interactions (1, 10–12). Some of these complexities can be captured by genome-scale metabolic models (GEMs) that allow rapid query of metabolic complexities through simulations of metabolic flux states, knockout experiments, multistrain metabolic comparisons, and calculation of metabolic characteristics (13, 14). Alternatively, coarse-grained modeling of metabolism attempts to peer beyond the detailed complexity and discover the general principles governing the biological systems of interest (15–17). In the present work, we took guidance from a coarse-grained model proposed in Escherichia coli coupled with genome-scale analyses of S. aureus transcriptional regulation and metabolism to uncover a similar staphylococcal system that balances resource allocation between carbon and nitrogen metabolism (15, 17).

Biological trade-offs represent an optimization frontier, where the cell must strike a balance between its multiple objectives and their limitations (15, 18). Signatures of these balancing acts can be found in transcriptomes and become apparent when their architecture is viewed at the systems level (19). We previously described one such trade-off and its transcriptional imprint using independent data sets from Gram-negative E. coli and Gram-positive S. aureus, in which a balance was observed between genes regulated by stress-associated sigma factors and growth-associated translation machinery (20, 21). Here, we expand significantly beyond those observations to describe a trade-off between carbon and nitrogen metabolism in strains of the globally disseminated, hypervirulent S. aureus USA300 lineage.

We first greatly expanded on our previous model of transcriptional regulation in USA300 strains to incorporate all publicly available RNA sequencing data from the Sequence Reads Archive (SRA) (21). Models were then generated by applying independent component analysis (ICA), which calculates independently modulated sets of genes (iModulons) and their activities present in the input RNA sequencing samples. iModulons represent sources of signals in the expression data, with transcriptional regulators being the most common source. Our model showed that the activities of two global metabolic regulators, CcpA and CodY, which play critical roles in central carbon and nitrogen metabolism, respectively, are negatively correlated against one another. This negative correlation pointed to a condition-specific reallocation of resources toward different metabolic subsystems. GEMs fitted with metabolomics data confirmed the inferences made from the transcriptomic data. Furthermore, GEMs revealed specific metabolic interfaces where coordination of metabolism by the two regulators is required for optimal biomass production, including glutamate dehydrogenase and the folate cycle. Placing genes from CodY and CcpA-associated iModulons onto the metabolic map demonstrated that they did not share any metabolic reactions but coregulated expression of a gene encoding ribosome hibernation factor. In light of these observations, we propose a model whereby CcpA and CodY coordinate gene expression for carbon metabolism, nitrogen metabolism, and translation, thus modularly controlling protein production at specific stages.

## RESULTS

### Expanding the USA300 iModulons using RNA sequencing data from the SRA database.

Our previous work outlined 29 iModulons for USA300 strains that were generated from 108 in-house RNA sequencing data (21). To expand the previous iModulons coverage of the TRN, we took advantage of the rapidly growing, publicly available S. aureus RNA sequencing samples (see Fig. S1 in the supplemental material). We queried the Sequence Reads Archive (SRA) for all available USA300-specific RNA sequencing data and combined it with 64 newly generated samples. Of the 576 sequencing samples available, 385 passed the stringent quality control/quality assurance (QC/QA) pipeline and were therefore incorporated into the new model (see Materials and Methods). The final set of samples contained data from at least 7 different USA300 isolates, 4 growth phases (exponential, stationary, biofilm, and infection), and 10 base media (Fig. S2).

10.1128/msystems.00480-22.1 FIG S1 Accumulation of publicly available S. aureus RNA sequencing data in SRA. Download FIG S1, EPS file, 0.8 MB. Copyright © 2022 Poudel et al. 2022 Poudel et al. https://creativecommons.org/licenses/by/4.0/ This content is distributed under the terms of the Creative Commons Attribution 4.0 International license.

10.1128/msystems.00480-22.2 FIG S2 Distribution of sample metadata used to build the USA300 model. The sample distribution is based on SRA metadata and manual curation from linked publications, if available. Download FIG S2, EPS file, 0.8 MB. Copyright © 2022 Poudel et al. 2022 Poudel et al. https://creativecommons.org/licenses/by/4.0/ This content is distributed under the terms of the Creative Commons Attribution 4.0 International license.

**FIG 1:**
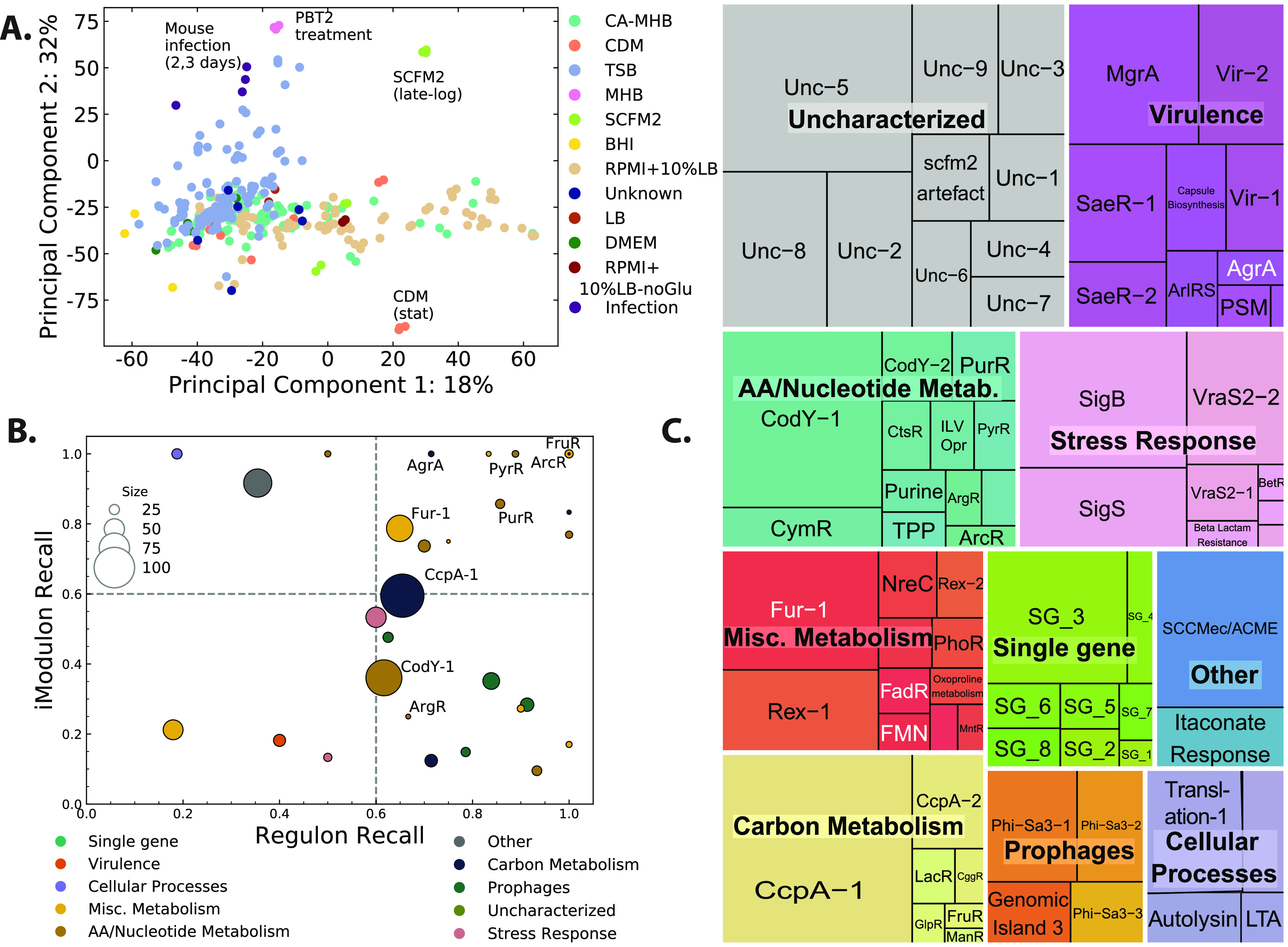
Updated iModulons for USA300 strains. (A) Principal-component analysis (PCA) of 385 RNA sequencing samples from diverse growth conditions that were used to generate the expanded USA300 iModulons. The log TPM of each sample was normalized to project-specific control conditions to reduce signal from batch effect before projecting them onto the principal components. (B) Association between iModulons and previously published regulons. iModulon recall represents the fraction of iModulon genes that were present in the regulon, while regulon recall represents the fraction of regulon genes that were present in the iModulon. iModulons that have high iModulon recall and high Regulon recall (e.g., PyrR, PurR) have very high overlap in gene content. (C) TreeMap of iModulon names, sizes (gene content), and types in the current model after manual curation. The size of the boxes correspond to the number of genes in the iModulon. stat, stationary; exp, exponential; TSB, tryptic soy broth; SCFM2, synthetic cystic fibrosis medium 2; CDM, chemically defined medium; TPM, transcripts per million.

Before applying ICA, we normalized the log-transformed transcripts per million (log-TPM) data to a project-specific control condition. This reduced batch-specific variation in the data and reduced the presence of iModulons not associated with biological signals. Principal-component analysis (PCA) of the log-TPM data showed that normalized samples tended to cluster with media types and growth phases rather than by data source (Fig. 1a). For example, data from S. aureus grown to late-log phase in SCFM2 (synthetic cystic fibrosis sputum medium 2) and to stationary phase in chemically defined medium (CDM) did not cluster together, despite being from the same bioproject.

Application of ICA to this normalized expression data resulted in 76 independent components, and genes with high absolute weightings within each component were assigned to a corresponding iModulon. These enriched iModulon genes were then compared with existing literature of predicted regulons in S. aureus. Those iModulons that had significant overlap with other predicted regulons were named after the associated regulator (Fig. 1b). Last, some iModulons with no known regulators but associated with other biological processes (e.g., prophages, translation) were manually curated. In total, we labeled 60 of the 76 iModulons with either a regulator or a biological process (Fig. 1c). The remaining uncharacterized iModulons represent signals in the S. aureus transcriptome with currently unknown origins, thus providing a roadmap to discovery of missing parts of the known TRN. In addition to the structure of each iModulon, the activities of each of the 76 iModulons in the 385 input samples were also calculated. The activity represents the role of each iModulon (and the associated regulator, if known) in shaping the transcriptome in the given sample. Higher iModulon activity represents a higher expression level of genes with positive weightings in the iModulon and lower expression of genes with negatively weighted genes (20).

### CcpA and CodY iModulon activities highlight balance of carbon and nitrogen metabolism.

**FIG 2:**
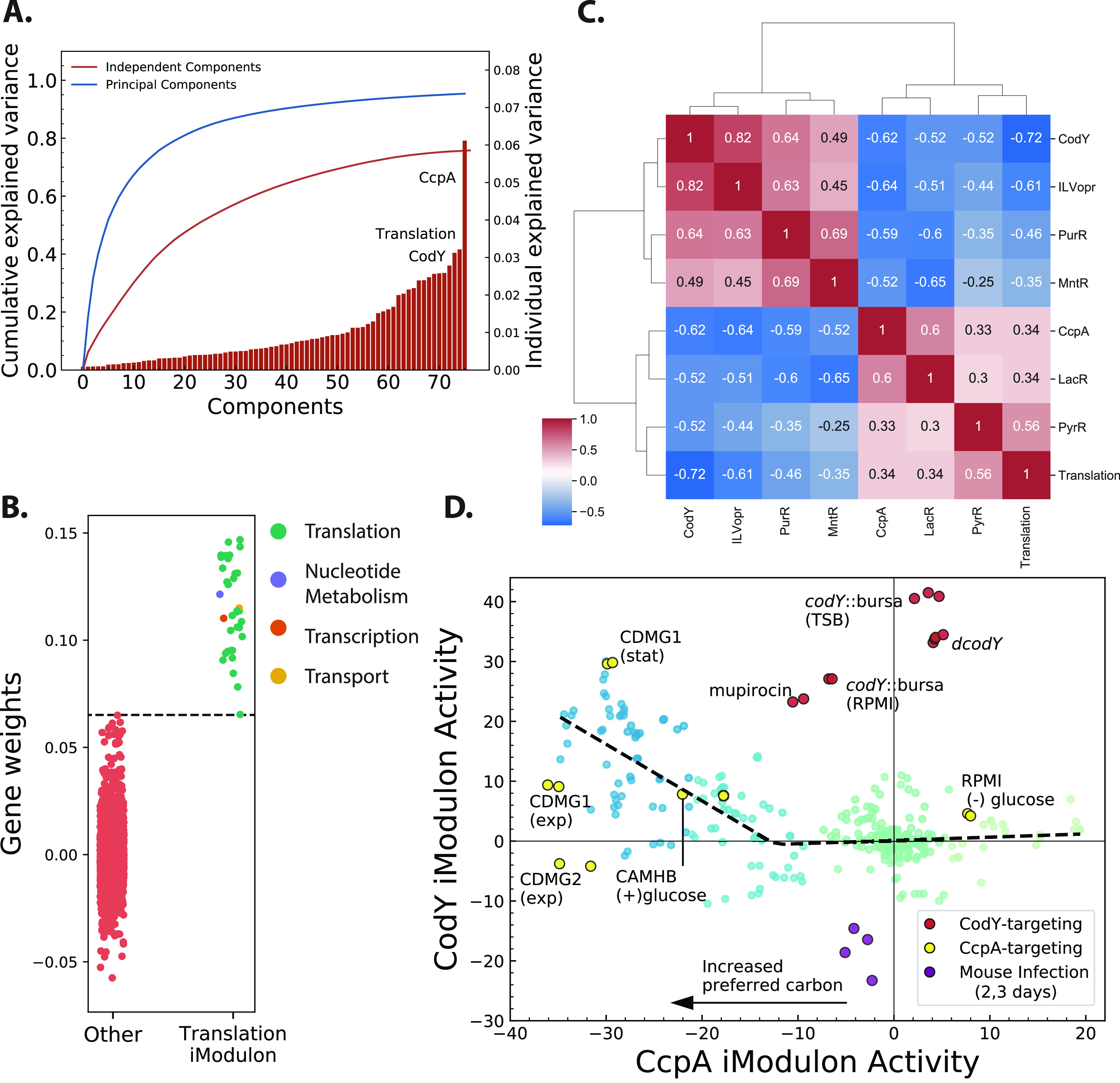
Coordination of metabolic iModulons in USA300 strains. (A) Explained variance of each of the iModulons; CcpA, Translation, and CodY iModulons explain the most variance in the transcriptome data. (B) Weightings of all the genes in the component associated with the Translation iModulon. Genes enriched in the iModulon (right column) are almost entirely part of the translation COG category. (C) Clustermap based on Pearson correlation coefficient of iModulon activities highlights potential coordination between various regulators. (D) Activity of CcpA and CodY iModulons across all USA300 samples shows biphasic association with close correlation in low CcpA activity conditions (blue markers) and little correlation otherwise (green markers). Large changes in CodY activity induced by genetic disruption of the *codY* gene or by mupirocin treatment does not alter CcpA activity (red markers). However, decrease in CcpA activity leads to increase in CodY activity (yellow markers). This asymmetric relationship suggests that CcpA works upstream of CodY. stat, stationary; exp, exponential; TSB, tryptic soy broth.

Cumulatively, the 70 iModulons captured ~70% of the variance in the input transcriptomic data. The CodY-1, CcpA-1 (henceforth referred to as simply CodY and CcpA iModulons, respectively), and Translation iModulons explained the most variation in the data (Fig. 2a). CcpA is the catabolite repressor protein in *Firmicutes* that represses genes involved in alternate carbon utilization, as well as other central carbon metabolic pathways, such as the tricarboxylic acid (TCA) cycle, in the presence of high concentrations of glucose. CodY, on the other hand, globally represses the genes required for amino acid biosynthesis in response to high branched-chain amino acid (BCAA) or GTP concentrations. Lastly, the Translation iModulon almost entirely consists of ribosomal genes (e.g., *rplK*, *rplA*, etc.) and genes involved in translation, such as *infA* and *fusA*, which encode translation initiation factor IF-1 and elongation factor G, respectively (Fig. 2b). This iModulon has been enriched in almost all bacteria and archaea for which iModulons have been calculated (20, 22–25).

Interestingly, the activities of these three iModulons were highly correlated across all samples (Fig. 2c) and formed a large cluster, along with other metabolic iModulons (Fig. S3). Along with CodY, CcpA, and Translation iModulons, activities of ILVopr (iModulon containing the operon with isoleucine, leucine, and valine biosynthesis genes), MntR, LacR, and PurR iModulons were also highly correlated. The correlation of CcpA with LacR simply reflects the catabolite repression of lactose utilization genes by the regulator CcpA. Similarly, the ILV operon is regulated globally by CodY and locally by leucine attenuator (26). This multilayer regulation likely explains why this operon formed its own iModulon whose activity was closely correlated with CodY. The MntR iModulon contains genes required for manganese uptake, and its coordinated activity with CcpA confirms the association of manganese concentration with glycolytic flux (27).

10.1128/msystems.00480-22.3 FIG S3 iModulon activity clustering. The iModulon activities formed distinct clusters indicating coordinated gene regulation. The highlighted individual clusters are remapped in the smaller clustermaps. Download FIG S3, EPS file, 4.6 MB. Copyright © 2022 Poudel et al. 2022 Poudel et al. https://creativecommons.org/licenses/by/4.0/ This content is distributed under the terms of the Creative Commons Attribution 4.0 International license.

The correlated activity of CcpA and CodY iModulons suggested that S. aureus carefully coordinates its central carbon and nitrogen metabolism (Fig. 2d). Close examination of the activities of these two iModulons showed a biphasic relationship. In conditions with preferred carbon sources, and therefore low CcpA iModulon activity, CodY activity generally increased. This effect was observed when glucose was added to both a complex medium (cation-adjusted Mueller-Hinton broth, or CA-MHB) and to a defined medium (chemically defined medium, or CDM1). Other conditions without explicitly controlled glucose levels that showed low CcpA activity still had concomitant high CodY activity, suggesting that this effect was not glucose specific. In conditions with already low CodY activity, however, removal of glucose (RPMI^−^ glucose; substituted with maltose) did not lead to further change in CodY activity, creating the second phase of the trade-off plane.

On the other hand, an increase in CodY iModulon activity did not necessarily lead to decrease in CcpA activity (Fig. 2d, red markers). Samples from several projects where the *codY* gene was interrupted showed minimal effect on CcpA iModulon activity. These samples fell well outside the CcpA-CodY trade-off line (Fig. 2d, gray dashed lines). Similar effects can also be observed in samples treated with subinhibitory concentrations of mupirocin. Mupirocin activates the stringent response in S. aureus, which leads to conversion of GTP to ppGpp and subsequent derepression of the CodY regulon (28). As change in CcpA activity leads to change in CodY activity but not necessarily vice versa, these data suggest that CcpA works “upstream” of CodY.

### Metabolic modeling supports coupling of CcpA and CodY activities.

To independently confirm the metabolic interaction between CodY and CcpA, we used a previously published USA300 strain-specific genome-scale metabolic model (GEM) (29). GEMs are curated mathematical models of an organism's metabolic network that can be used to simulate, study, and design the metabolic pathways using a wide range of constraints-based reconstruction and analysis (COBRA) tools (14, 30).

**FIG 3:**
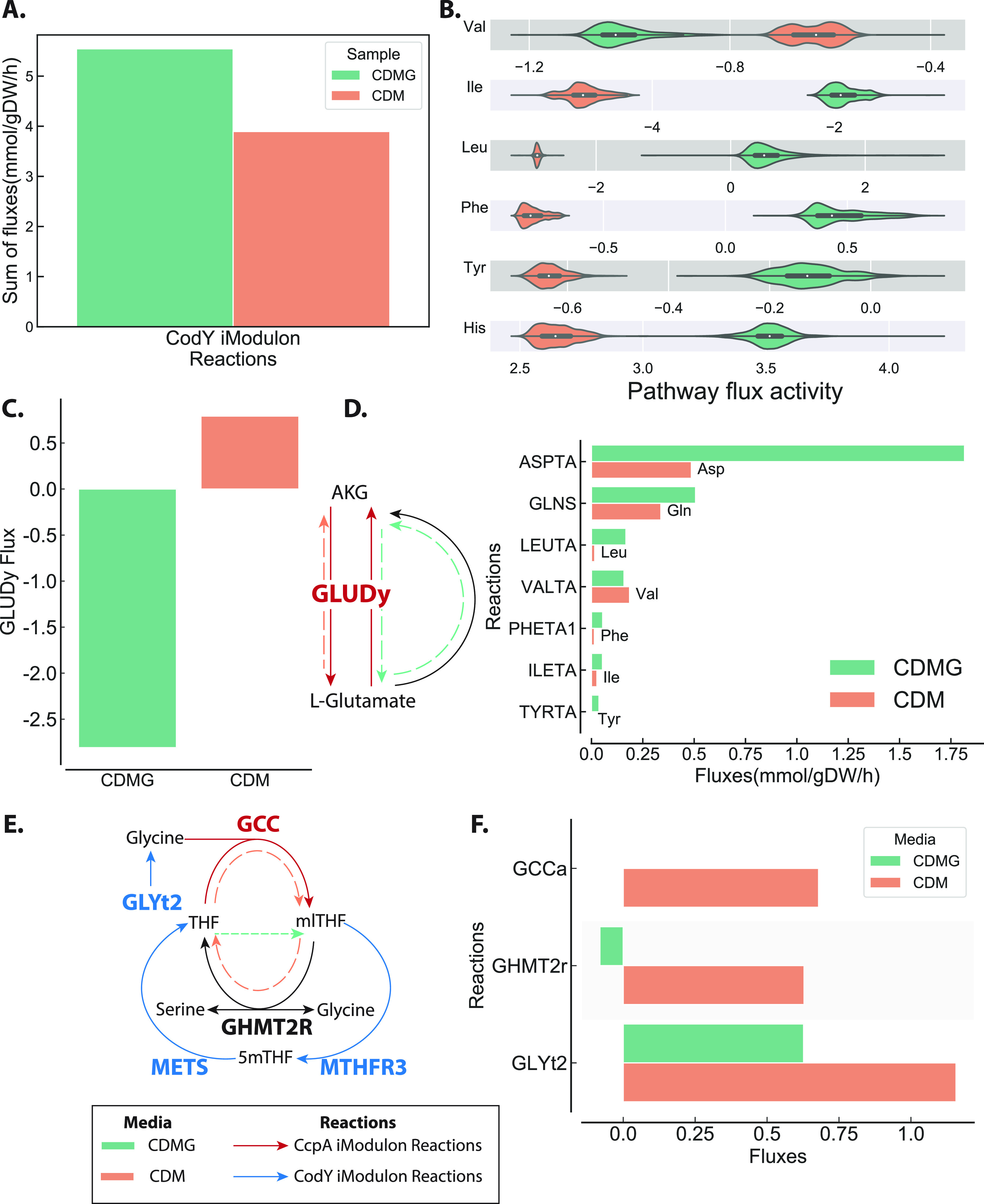
Metabolic modeling shows coordination of CcpA and CodY fluxes. (A) The solution of space of the models with CDM- and CDMG-specific metabolic constraints was sampled. The sum of sampled fluxes through CodY and CcpA reaction shows increased flux through CodY in CDMG. (B) Sampled fluxes through several amino acid biosynthetic pathways also show increased flux in CDMG. Definition of the biosynthetic pathways is found in Materials and Methods. (C) Flux through GLUDy reaction changes direction when glucose is added; alpha-ketoglutarate (AKG) is converted to l-glutamate in CDMG and vice versa in CDM. l-Glutamate is then utilized in biosynthesis of other amino acids in CDMG regenerating AKG. (D) Amino acids generated by accepting amine groups from l-glutamate, coupling the CcpA reaction GLUDy with amino acid-generating CodY reactions. (E) Metabolic map of folate cycle where CcpA and CodY regulated metabolism intersect. (F) Simulated flux through reactions in folate cycle in CDM and CDMG shows alternate metabolic reactions used in each medium. In CDM, reactions that consume glycine (GCC and GHMT2r) to interconvert between THF and methyl-THF (mlTHF). In CDMG, glycine is instead generated by GHMT2R running in reverse.

One such method, parsimonious flux balance analysis (pFBA), can be used to calculate the metabolic flux state that optimizes a phenotype while minimizing total metabolic flux in a given condition (13, 31). pFBA thus represents a parsimonious use of the metabolic proteome. Here, we used pFBA to determine the metabolic flux states that maximize S. aureus biomass production given the measured uptake and secretion rates of various amino acids and sugars in chemically defined medium (CDM) and CDM with glucose (CDMG) (10). In agreement with increased CodY iModulon activity in CDMG, total flux through reactions catalyzed by enzymes that are encoded in CodY iModulon genes (“CodY reactions” for short) increased from 3.9 mmol/gram dry weight (gDW)/h to 5.5 mmol/gDW/h in the presence of glucose (Fig. 3a). The total sum of fluxes through CcpA reactions in our pFBA simulations did not change drastically between CDM and CDMG (Fig. S4). Many CcpA fluxes simply reversed directions (e.g., reactions involved in the tricarboxylic acid cycle), some of which are described in detail in the next section.

10.1128/msystems.00480-22.4 FIG S4 Sum of CcpA reaction fluxes in CDM and CDMG. pFBA solutions from simulating metabolism in CDM and CDMG media showed little change in total absolute flux going through CcpA reactions. Though the total magnitude of the total flux did not change much, many CcpA reactions reversed their overall flux direction in CDMG. Download FIG S4, EPS file, 0.6 MB. Copyright © 2022 Poudel et al. 2022 Poudel et al. https://creativecommons.org/licenses/by/4.0/ This content is distributed under the terms of the Creative Commons Attribution 4.0 International license.

pFBA, however, gives an exact optimal solution and therefore does not account for variations or errors in input uptake data. We addressed this issue by sampling the CDM- and CDMG-specific models, which give a distribution of feasible fluxes in each of the respective conditions. We then mapped the condition-specific flux distributions to various amino acid biosynthetic pathways. For simple interpretation, we excluded amino acids that serve as intermediates for biosynthesis of other amino acids (e.g., glutamine, glutamate, and serine) and included only those amino acids for which unique biosynthetic pathways could be defined (see Materials and Methods). Confirming pFBA analysis, 5 out of the 6 amino acid biosynthetic pathways had increased flux in CDMG compared to CDM (Fig. 3b). The results of these two TRN-agnostic metabolic modeling methods are in agreement with our observation that CodY iModulon activity increases in the presence of glucose.

### Transcriptional coordination of CcpA and CodY is likely due to flux coupling at metabolic interfaces.

CcpA and CodY iModulons contained 110 and 86 genes, respectively. Most of these genes are involved in central carbon and amino acid metabolism. Despite the large iModulon sizes and close metabolic proximity of the regulated genes, the two iModulons did not share any genes encoding metabolic enzymes. The correlation in iModulon activities, however, suggested that CcpA reactions and CodY reactions must be coordinated at a metabolic level. Using the USA300 GEM, we looked for this coordination at the metabolite interface between CcpA and CodY reactions, i.e., metabolites that are involved in both CcpA and CodY reactions. We found these metabolic interfaces by systematically identifying all metabolites in USA300 GEM that can be found in both CodY and CcpA reactions. After taking out “nonspecific” metabolites and cofactors (e.g., ATP, H_2_O, NADH, etc.), we were left with 22 metabolites at the interface (Table S1). While some of these metabolites, such as pyruvate, glutamate, and oxaloacetate, are expected, as they play a crucial role in both carbon and nitrogen metabolism, other metabolites, such as *N*-succinyl-2-l-amino-6-oxoheptanedioate and tetrahydrofolate (THF), are less understood in the context of this trade-off. To further understand how a change in simulated flux through CcpA and CodY reactions in CDM and CDMG altered these key metabolic interfaces, we mapped the pFBA solution fluxes from each media to the reactions around two of these interfaces, glutamate and methyl-THF.

10.1128/msystems.00480-22.8 TABLE S1 Interface metabolites. Metabolites at the interface of CcpA and CodY iModulons and the CcpA and CodY reactions they are involved in. Download Table S1, CSV file, 0.00 MB. Copyright © 2022 Poudel et al. 2022 Poudel et al. https://creativecommons.org/licenses/by/4.0/ This content is distributed under the terms of the Creative Commons Attribution 4.0 International license.

The glutamate-alpha-ketoglutarate (αkg) link is a closely studied interface in S. aureus that connects amino acid and central carbon metabolism (5, 10). The main enzyme at the interface, glutamate dehydrogenase (GLUDy), reversibly interconverts αkg and glutamate and is encoded by the *gudB* gene, a constituent of the CcpA iModulon. However, this interconversion also acts as an amine group donor or acceptor to 3 CcpA reactions and 8 CodY reactions (Table S1). In glucose-free CDM, pFBA solution was consistent with a previous observation showing proline is converted to αkg via glutamate and eventually fuels gluconeogenesis (10). However, in CDMG, the flux through GLUDy changes direction and catalyzes conversion of αkg to glutamate instead (Fig. 3C). This makes up ~98% of total flux that consumes αkg. The glutamate, in turn, acts as an amine group donor for biosynthesis of various amino acids and accounts for ~80% of total flux generating αkg in CDMG (Fig. 3D). pFBA solution of this interface therefore shows that in the absence of glucose, the GLUDy reaction converts glutamate to αkg to fuel gluconeogenesis, but in the presence of glucose, it converts αkg to glutamate to fuel amino acid biosynthesis.

The folate cycle represents another metabolic interface of CcpA and CodY reactions. The folate cycle is required for one-carbon metabolism, nucleotide biosynthesis, and amino acid metabolism, and the pathway leading up to the cycle is the target of sulfonamide-class antibiotics (32). The folate cycle consists of 2 CodY reactions, MTHFR3 and methionine synthase (METS), and one CcpA reaction, GCCabc (glycine cleavage complex) (Fig. 3E). In CDM, THF is converted into 5,10-methylenetetrahydrofolate (mlTHF) by the GCCabc reaction, which cleaves glycine in the process (Fig. 3F). THF is then regenerated from mlTHF by the GHMT2r reaction, which also consumes glycine and generates serine. This consumption of glycine in the folate cycle by the CcpA reaction is coupled with increased transport of glycine by CodY-regulated GLYt2. However, in CDMG, where CcpA iModulon activity is low, there is no flux through the CcpA reaction, GCCabc. Instead, GHMT2r runs in “reverse” to convert THF from mlTHF, consuming serine and generating glycine instead. Together, combining iModulon structure with metabolic simulation demonstrates how S. aureus coordinates flux through CcpA and CodY iModulon reactions at these key metabolic interfaces, despite not sharing any genes at the regulatory level.

### Expression levels of translation-associated genes are responsive to CcpA and CodY activities.

**FIG 4:**
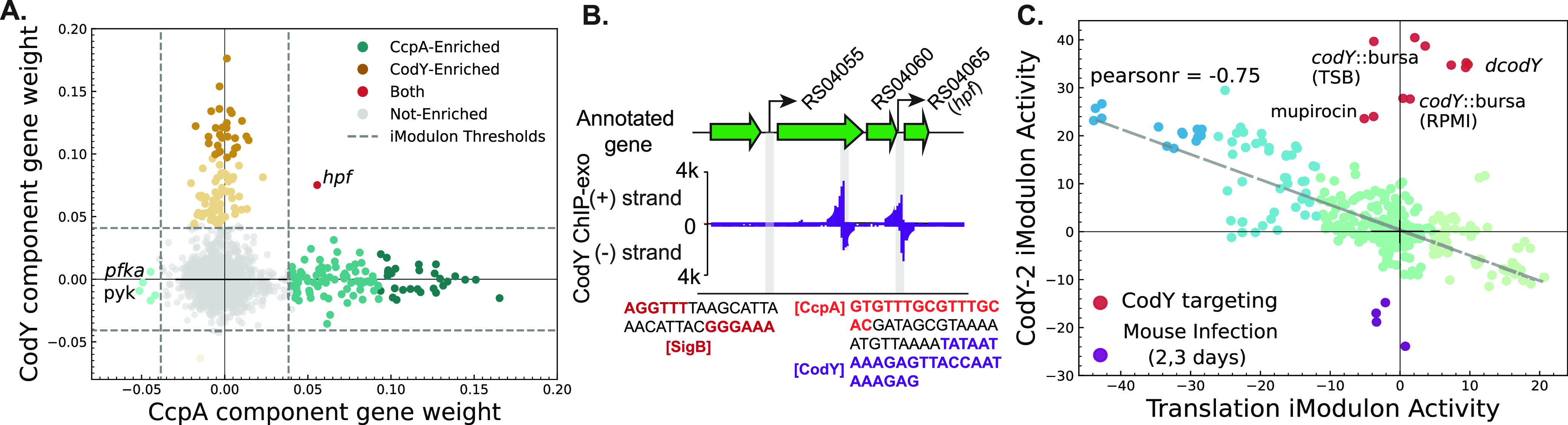
Coordination of translation with metabolism. (A) Gene weights in CcpA and CodY iModulon-associated components shows that only the *hpf* gene is a part of both iModulons. (B) Upstream region of hpf gene with its two alternative transcription start sites. Two CodY binding sites were detected by ChIP-exo (purple bars). The previously recognized SigB(red) and CodY(purple) binding sites and newly proposed CcpA (orange) binding site are highlighted. (C) The negative correlation between CodY and Translation iModulon activity suggests coordination of metabolism and translation in S. aureus. As with CcpA and CodY association, samples where CodY activity is altered by genetic disruption of *codY* or by treatment with mupirocin (in red) break this coordination. Other samples are colored in green-blue palette based on Translation iModulon activity.

While CcpA and CodY iModulons do not share any metabolic genes, *hpf*, which encodes ribosomal hibernation-promoting factors (HPFs), is a gene found in both iModulons (Fig. 4a). HPF is a small peptide that dimerizes 70S ribosomal subunits to form inactive 100S subunits (33, 34). It plays an important role in stress response and nutrition limitation and protects ribosomal pools from degradation (35–37). Previous studies in S. aureus have shown that SigB and CodY regulate *hpf* expression in response to heat and nutritional stress (35). iModulon structure confirms the role of the CodY and suggests an additional layer of control by CcpA.

Chromatin immunoprecipitation coupled with exonuclease treatment (ChIP-exo) data from our previous work found two CodY binding sites in the regulatory region of the *hpf* gene (Fig. 4b) (38). To confirm the role of CcpA in *hpf* expression, we searched for the catabolite repressor element motif (WTGNNARCGNWWWCAW) from Bacillus subtilis in the same region (39). A matching motif was found in the region between the two CodY binding peaks (false-discovery rate [*q*] = 0.00905). This architecture, with two CodY binding sites flanking the CcpA binding site, is also found in the regulatory region of the B. subtilis BCAA operon, where both regulators contribute to the expression of the operon genes (40). The signal from expression data and the presence of binding motifs suggests that CcpA regulates *hpf* along with the previously identified regulators, CodY and SigB.

In addition to coordinated regulation of the translation-associated *hpf* gene, CodY activity was also strongly correlated with Translation iModulon activity. In contrast, CcpA and Translation iModulon activities showed little correlation between them (Fig. S5). Similar to CcpA and CodY activity correlation, *codY* knockout and stringent response activation by mupirocin also disrupted correlation with Translation iModulon (Fig. 4c). This also suggested that the signal controlling Translation iModulon gene expression also works upstream of CodY, as interruption of CodY had little effect on Translation iModulon activity. While the coordination of the two iModulon activities is apparent, we were unable to further interrogate the nature of this relationship since the signal behind the Translation iModulon is yet to be identified.

10.1128/msystems.00480-22.5 FIG S5 The iModulon activities of CcpA and translation iModulons showed little correlation. Download FIG S5, EPS file, 1.1 MB. Copyright © 2022 Poudel et al. 2022 Poudel et al. https://creativecommons.org/licenses/by/4.0/ This content is distributed under the terms of the Creative Commons Attribution 4.0 International license.

## DISCUSSION

**FIG 5:**
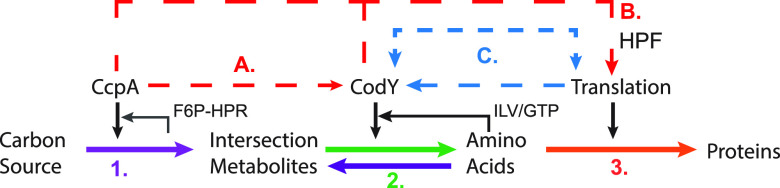
Proposed coarse-grained model of protein biosynthesis regulation in S. aureus. The solid lines represent the parts of the protein synthesis pathway controlled by CcpA (purple) and CodY (green). The dashed lines represent new proposed roles of these regulators in coordinating carbon and nitrogen metabolism (A) and linking metabolic gene expression with expression of translation-associated proteins (B, C). At the regulatory level, CcpA and CodY activities are coordinated at high glucose conditions. Similarly, CodY, but not CcpA, activity is correlated with the activity of Translation iModulon. CcpA and CodY also feed forward to regulate the expression of the *hpf* gene, which encodes a hibernation factor that can alter the fraction of active ribosomes in the cell. Through this mechanism, S. aureus may be changing the concentration of the active ribosome in response to metabolic cues.

Based on the data presented here, we propose a coarse-grained model of transcriptional regulation of metabolism involved in protein synthesis in S. aureus USA300 strains (Fig. 5). It is motivated by the model of proteome coordination in E. coli and extends its principles to nonmodel pathogenic organisms (15). The coarse-grained model simplifies metabolism underlying protein synthesis into three steps, (i) the generation of precursors from the carbon source, (ii) biosynthesis of amino acids from precursors or direct transport from the medium, and (iii) synthesis of peptides from amino acids via translation. The generation of precursors from carbon sources is largely regulated by CcpA (Fig. 5, purple arrow). CcpA represses alternate carbon sources (including amino acids such as proline, glutamine, and aspartate) in the presence of preferred carbon (such as glucose) and regulates other key aspects of central metabolism, such as gluconeogenesis and the TCA cycle, that are necessary to generate various precursors (1, 10, 41–43). The precursors in our model are represented by the metabolites at the CcpA-CodY interface derived from the USA300 GEM (see Table S1 in the supplemental material). These precursors are then converted to amino acids via CodY-regulated gene products (green arrow) and polymerized by ribosomes into proteins (light blue arrow) (38, 43, 44).

Our analysis suggests that S. aureus USA300 strains coordinate their CcpA and CodY activity to regulate carbon and nitrogen flow through the system (dashed orange arrow). Metabolic modeling in CDMG shows increased flux through amino acid biosynthetic reactions compared to CDM. The results of this TRN-agnostic metabolic model agree with the increased CodY activity in CDMG and other glucose-containing media. Additionally, we also found a feed-forward regulation whereby CcpA and CodY control the expression of the gene encoding the HPF protein, which sequesters ribosomes into inactive 100S forms, suggesting a mechanism by which translation is coordinated with the metabolic state of the cell (dashed red arrows) (33, 35).

Last, the activity of the Translation iModulon is also closely correlated with CodY activity, which may act as an additional layer of coordination between metabolism and translation (dashed blue arrows). However, we have yet to identify the signal or regulator controlling the Translation iModulon activity, and therefore, the source of this concomitant change in expression along with CodY is unclear. rRNA expression is regulated by ppGpp during the stringent response, which can be activated by mupirocin treatment (28, 45). We therefore expected mupirocin to also have an effect on Translation iModulon activity, but we found that while CodY activity increased in response to mupirocin as expected, there was minimal change in Translation activity (Fig. 4c). This suggests that stringent response, at least when induced by mupirocin treatment, does not play a major role in the expression of Translation iModulon genes.

Despite close coordination of metabolic flux at different interfaces between CcpA and CodY reactions, it is still not clear how CcpA and CodY activities are coordinated. In E. coli, Kochanowski et al. have observed similar coordination between anabolic and catabolic fractions of metabolism (46). The authors attributed active regulation by Crp and passive changes in metabolic fluxes in response to change in metabolite concentrations as the source of the coordination. Other nonmetabolic constraints could also play a role in this coordination. The agreement between our metabolic model and the iModulon activities only arises after the metabolic model is constrained using experimentally measured amino acid uptake rates. This suggests that other nonmetabolic factors may be leading to differential amino acid uptake rates in glucose media and subsequently leading to the change in CodY activity. In this sense, the problem may be similar to the inability of unconstrained metabolic models to predict overflow metabolism, which has been postulated to result from other nonmetabolic constraints, such as proteome and membrane real estate allocation (47, 48). The root of this relationship, whether metabolic or nonmetabolic in nature, is yet to be determined.

The analysis of the coarse-grained model of metabolic gene regulation presented here was enabled by a computable model of TRN. iModulons enable us to query the TRN at multiple scales, giving insights into TRN from single-gene membership level to global coordination of regulators. By modularizing the TRN, our analysis enabled us to unravel complex regulatory and metabolic interactions to understand regulation of the central metabolism one regulator at a time. This modularization can also be used to continually expand on the presented model. For example, our previous works have shown that Translation iModulon activity in E. coli and S. aureus is closely correlated with stress-associated alternate sigma factors (20, 21). This points to a possible entry point for incorporation of general stress response with metabolism and protein synthesis. Similarly, we have also found that both PyrR and PurR activity is correlated with CodY and CcpA, which may provide insights into the regulation of nucleotide biosynthesis in response to carbon or nitrogen availability. While we mainly focused on 3 iModulons, CcpA, CodY, and Translation, the current model contains 76 total iModulons, each of them rich with information about transcriptional regulation and physiology of S. aureus. We thus provide a conceptual framework for overall coordination of metabolism in S. aureus and approaches to systematically expand and detail the model proposed.

## MATERIALS AND METHODS

### Strains and growth conditions.

The S. aureus USA300 isolate, LAC, and its derivative, JE2, were used to collect the new RNA sequencing data in this study. The complete description and condition for each of the samples can be found in Table S2 in the supplemental material. For RNA sequencing from knockout samples, isolates from the Nebraska Transposon Mutant Library were utilized (49). Unless specified otherwise, samples were grown in duplicates in 20 mL of respective media until they reached the optical density at 600 nm (OD_600_) of 0.5. Three milliliters of culture was harvested and immediately mixed with 6 mL of Qiagen RNAprotect bacteria reagent and incubated at room temperature for 5 min. The supernatant was decanted after the samples were centrifuged for 10 min and 17,500 rpm. The remaining cell pellets were stored at −80°C until they were prepared for RNA extraction.

10.1128/msystems.00480-22.9 TABLE S2 Gene iModulon membership. Enriched iModulons, their gene contents, and the gene descriptions. Download Table S2, CSV file, 0.3 MB. Copyright © 2022 Poudel et al. 2022 Poudel et al. https://creativecommons.org/licenses/by/4.0/ This content is distributed under the terms of the Creative Commons Attribution 4.0 International license.

### RNA extraction and sequencing.

Total RNA was isolated from the cell pellet in the Qiagen RNeasy minikit columns by following vendor procedures. An on-column DNase treatment was performed for 30 min at room temperature. The rRNA was removed using RiboRid protocol, as described before (50). RNA was quantified using a NanoDrop and quality assessed by running an RNA nanochip on a bioanalyzer (Agilent, CA). A Swift RNA library kit was used following the manufacturer’s protocol to create sequencing libraries.

### Preparing RNA sequencing data for iModulon calculation.

The iModulons were calculated from publicly available RNA sequencing data from SRA and the newly collected data in this study using PyModulon python package (51). The steps used to calculate the iModulons described here were all completed using this package. All RNA sequencing data labeled with S. aureus taxonomic ID were downloaded and manually curated to obtain only the samples that were from USA300 isolates. Raw fastq files from curated samples were downloaded, trimmed with TrimGalore, and then aligned to the USA300 TCH1516 genome (GenBank accession nos. NC_010079, NC_012417, and NC_010063) using Bowtie2 (52). QC/QA stats were collected on each sample using MultiQC, and samples that did not pass the QC thresholds (e.g., low read depth, low correlation between replicates, missing metadata) were discarded (53). Transcripts per million (TPM) were calculated from the remaining high-quality RNA sequencing samples. TPM were log transformed and normalized to a control condition within the same bioproject.

### Calculating iModulons from RNA sequencing data.

Scipy’s implementation of FastICA was applied to log-transformed and normalized TPM data to generate independent components (ICs) and their activities (54, 55). Unlike other decomposition methods, ICA requires the number of dimensions to be calculated as an input. Therefore, various models with different dimensionality were created, and the one that maximized regulatory iModulons and minimized single-gene iModulon was chosen (56). The iModulons were then automatically annotated if they overlapped significantly with a curated list of known or predicted regulons and genomic features (e.g., prophages, SCCMec, ACME, etc.) in S. aureus. Other iModulons, such as “Translation” or “Autolysin,” were manually annotated, as all genes contained within the iModulons have a single function. The gene content of iModulon can be found in Table S2.

### Genomic-scale modeling of S. aureus USA300 metabolism.

The USA300-specific genome-scale model (GEM) iYS854 was used for all metabolic simulations in the paper. The exchange rates of amino acids, glucose, ammonium, and acetate were adjusted to constrain the model to CDM- or CDMG-specific conditions as described in detail before (29). Briefly, the uptake or secretion rate for each metabolite from Halsey et al. was normalized by growth rate to get the growth-adjusted solute uptake rate (10). The exchange rates were then constrained to ±15% of uptake and exchange rate to account for variance in the data.

Once constrained, the model was then used to calculate the flux of each medium using pFBA as implemented in the COBRApy package (30, 31). To get the CodY iModulon-specific flux, genes in the CodY iModulon were first mapped to metabolic reactions using the gene product rule (GPR). The absolute values of fluxes from the pFBA solution for the CodY reactions were then summed to get the final CodY iModulon flux.

To calculate the valid amino acid biosynthesis pathway-specific flux distribution, the solution spaces of CDM- and CDMG-specific models were sampled 10,000 times using the artificial centering hit-and-run algorithm (57). Next, the reactions in each amino acid biosynthetic pathway were determined with the MinSpan algorithm (58). MinSpan calculates the set of shortest metabolic pathways that are linearly independent of one another and span the null space of the input model. Each independent pathway defines a mass-balanced set of reactions and therefore enables unbiased modularization of metabolism into biologically meaningful pathways. The sampled fluxes (*v*) can therefore be represented as linear weightings (α) of minspan pathways (*P*), where

v=P×α

The sampled fluxes were converted to pathway-specific weightings (pathway fluxes) using the minspan matrix. Pathways containing amino acid biosynthesis were manually curated, and only amino acid biosynthesis pathways that did not appear in multiple MinSpan pathways were used for analysis, as they can be easily interpreted and do not require analyzing linear combinations of multiple pathways.

Last, the interface metabolites were determined by comparing all metabolites that were involved in at least one CodY and one CcpA reaction. The common metabolites ADP, ATP, CO_2_, coenzyme A, H_2_O, hydrogen atom, sodium ion, NAD, NADH, NADP, NADPH, ammonium (NH4), and phosphate were excluded from this designation.

### Motif enrichment.

The 150 base pairs upstream of the *hpf* gene (USA300HOU_RS04065) were scanned for the CcpA motif (WTGNNARCGNWWWCAW) using find individual motif occurrence (FIMO) within the MEME suite (39, 59).

### Data and code availability.

All RNA sequencing data used in this work are available publicly at Sequence Reads Archive (SRA). The accession numbers for individual samples can be found in Table S2. The code used to create the model and generate all the figures in the paper can be found on GitHub (https://github.com/sapoudel/metabs-paper-code). An interactive version of the iModulon model is also available at iModulonDB (https://imodulondb.org/).

10.1128/msystems.00480-22.6 FIG S6 RNA sequencing quality control metrics. (a and b) Samples with less than 500,000 reads were filtered out (a), as were samples with Pearson correlations of less than 0.9 (b). Download FIG S6, EPS file, 1.0 MB. Copyright © 2022 Poudel et al. 2022 Poudel et al. https://creativecommons.org/licenses/by/4.0/ This content is distributed under the terms of the Creative Commons Attribution 4.0 International license.

10.1128/msystems.00480-22.7 FIG S7 iModulon postprocessing. (a) ICA models with different dimensionalities were created with OptICA. The model using 180 dimensions, which had the maximum number of robust components while still keeping single gene components to a minimum, was chosen as the final model. (b) Some iModulons were labeled based on data from gene knockout experiments. Here, the iModulon with lowest activity in delta *arlRS* strain was labeled as ArlRS iModulon. Download FIG S7, EPS file, 1.1 MB. Copyright © 2022 Poudel et al. 2022 Poudel et al. https://creativecommons.org/licenses/by/4.0/ This content is distributed under the terms of the Creative Commons Attribution 4.0 International license.

10.1128/msystems.00480-22.10 TEXT S1 Creating the iModulon model for TRN of S. aureus USA300 data. Detailed description of the steps taken to build the iModulon model of S. aureus USA300 strain. Download Text S1, DOCX file, 0.01 MB. Copyright © 2022 Poudel et al. 2022 Poudel et al. https://creativecommons.org/licenses/by/4.0/ This content is distributed under the terms of the Creative Commons Attribution 4.0 International license.
