# Supplementary material for: Coordination of CcpA and CodY Regulators in Staphylococcus aureus USA300 Strains
Source: mSystems. 2022 Nov 2;7(6):e00480-22. doi: 10.1128/msystems.00480-22 (PMC9765215; doi:10.1128/msystems.00480-22)
Supplement: TEXT S1 [file msystems.00480-22-s0001.docx]

**Supplementary Note: Creating the iModulon model for TRN of *S. aureus* USA300 data**

This section provides detailed information on the steps taken to build the iModulon model of *S. aureus* USA300 strains. The methods described in this section were developed previously in *E. coli*, which contains further details and explanations[[1,2]](https://paperpile.com/c/wHXH5s/8miz+C57x). We started the process by downloading metadata for all the available *S. aureus* RNA sequencing data in the Short Reads Archive (SRA). We then curated the metadata manually to separate samples from a USA300 lineage (e.g. TCH1516, LAC, FPR3757 etc) from all other strains. Fastq files associated with these samples were downloaded from SRA, trimmed with TrimGalore and aligned to the TCH1516 reference genome, including the two plasmids ( NC_010079, NC_012417, NC_010063) using Bowtie2[[3–5]](https://paperpile.com/c/wHXH5s/z9pR+bzSB+oUCv). The gene read counts were then determined using HTSeqCount with intersection-strict criteria. The counts were then normalized and transformed to create log2TPM[[6]](https://paperpile.com/c/wHXH5s/HM8w).

The quality of the alignment was checked using fastqc and any data failing ‘per base sequence quality,’ ‘per sequence quality score,’ ‘per base n content’, or ‘adapter content’ were dropped. We also dropped samples with less than 500,000 reads aligned to one of the known CDS in TCH1516 (**Figure S6a**). These QC stats were organized into a single metadata using MultiQC[[7]](https://paperpile.com/c/wHXH5s/nh41). Samples that had poor correlation with other samples or clustered with samples from different projects were also excluded. For all samples that passed these QC steps, we searched through online records including SRA, BioSample or linked publications to gather additional information such as base media, growth conditions, mutations etc. We discarded samples that had little or no metadata.

All samples were assigned to a specific project based on the source of the data i.e. all samples from the same BioProject or publication were assigned to the same project. The samples in all projects were checked for reproducibility by checking the Pearson correlation between log2TPM of replicates. Samples with no replicates or those with r-value less than 0.9 were excluded at this step (**Figure S6b**). For each project, we identified one reference or control condition. This condition was used to center the data, by subtracting the log2TPM value of the reference from all other conditions in the same project. This reduces iModulons associated with inter-project batch effects. It also sets the log2TPM and all iModulon activities in reference conditions to 0 which allows us to easily interpret activity of iModulons in other samples as fold change from the control. The final 385 samples that passed these QC/QA steps were used to calculate iModulons.

We applied FastICA implemented in the scikit-learn package to calculate the M and A matrix from the logTPM data[[8,9]](https://paperpile.com/c/wHXH5s/xs6I+jruE). FastICA was applied 100 times with random seed and identical components from each run (which may contain slightly different values) were identified after clustering with DBSCAN. Only components that appeared in each run were kept for further analysis. Unlike PCA, the number of components that ICA calculates is not fixed and is a required input in FastICA. Decomposing the transcriptome into too few components can lead to signals from multiple regulators being combined into one iModulon. On the other hand, too many components leads to over decomposition that results in iModulons with a single gene or iModulons with near 0 activity in all samples. To determine the ideal number of components, we used our previously developed OptICA method[[10]](https://paperpile.com/c/wHXH5s/sNjB). OptICA runs ICA with 10 to 340 components with 10 component increments as inputs. For each model, with different component number input, we checked the number of robust and single gene iModulons. For the final model, we chose iModulons calculated with 170 components as it maximized robust components while minimizing the number of single gene iModulons(**Figure** **S7a**).

Once the model with optimal dimensionality was identified, we annotated the iModulons. iModulons were first annotated by comparing the enriched genes in each component to other predicted regulons from regPRECISE and other literature sources(see ‘TRN’ object in the model)[[11]](https://paperpile.com/c/wHXH5s/ST36). iModulons with significant overlap with predicted regulons; significant overlap was defined as hypergeometric test p-value <0.05, precision >= 0.5 and coverage >= 0.2. However, we also manually curated iModulons as not all regulators have predicted regulons and ICA can predict iModulons that are associated with other biological features (e.g. plasmids, prophages, gene deletions etc). ‘Functional’ iModulons were named after the functions of enriched genes in them e.g. Translation, Autolysins and Beta Lactam Resistance. In cases where data from regulator deletion mutants were available, iModulons were named if they showed the highest change in activity in the mutants (**Figure S7b**).
